# Inter-Rater Agreement for Diagnosing Adenomyosis Using Magnetic Resonance Imaging and Transvaginal Ultrasonography

**DOI:** 10.3390/diagnostics13132193

**Published:** 2023-06-28

**Authors:** Johanna K. Andersson, Raffaella Pozzi Mucelli, Margit Dueholm, Susanne Fridsten, Aristeidis Grigoriadis, Stefano Guerriero, Francesco Paolo Leone, Lil Valentin, Thierry Van Den Bosch, Nikolaos Voulgarakis, Kristina Gemzell-Danielsson, Elisabeth Epstein

**Affiliations:** 1 Department of Women’s and Children’s Health, Karolinska Institutet and Liljeholmens Gynecological Clinic, 11794 Stockholm, Sweden; johanna.k.andersson@regionstockholm.se; 2 Department of Clinical Science, Intervention, and Technology (CLINTEC), Division of Radiology, Karolinska Institutet, 17177 Stockholm, Sweden; pozzina@gmail.com (R.P.M.); aristeidis.grigoriadis@regionstockholm.se (A.G.); nikolaos.voulgarakis@regionstockholm.se (N.V.); 3 Department of Abdominal Radiology, Karolinska University Hospital, 17176 Solna, Sweden; susanne.fridsten@regionstockholm.se; 4 Department of Gynecology, Aarhus University Hospital, 8200 Skejby, Denmark; dueholm@dadlnet.dk; 5 Department of Molecular Medicine and Surgery, Karolinska Institutet, 17177 Stockholm, Sweden; 6 Department of Obstetrics and Gynecology, University of Cagliari, Policlinico Universitario Duilio Casula, 09042 Monserrato, Italy; gineca.sguerriero@tiscali.it; 7 Biomedical and Clinical Sciences Institute L. Sacco and Department of Obstetrics and Gynecology, University of Milan, 20122 Milan, Italy; francesco.leone@asst-fbf-sacco.i; 8 Department of Clinical Sciences Malmö, Lund University, 22100 Lund, Sweden; lil.valentin@med.lu.se; 9 Department of Obstetrics and Gynecology, Skane University Hospital, 21428 Malmö, Sweden; 10 Department of Obstetrics and Gynecology, KU Leuven, 3000 Leuven, Belgium; thierry.vandenbosch@uzleuven.be; 11 Department of Women’s and Children’s Health, Karolinska Institutet and Karolinska University Hospital, 17176 Stockholm, Sweden; kristina.gemzell@ki.se; 12 Department of Clinical Science and Education, Karolinska Institutet, Södersjukhuset, 11883 Stockholm, Sweden; 13 Department of Obstetrics and Gynecology, Södersjukhuset, 11883 Stockholm, Sweden

**Keywords:** adenomyosis, ultrasonography, magnetic resonance imaging, inter-rater agreement

## Abstract

Our aim was to compare the inter-rater agreement about transvaginal ultrasonography (TVS) with magnetic resonance imaging (MRI) with regard to diagnosing adenomyosis and for assessing various predefined imaging features of adenomyosis, in the same set of women. The study cohort included 51 women, prospectively, consecutively recruited based on a clinical suspicion of adenomyosis. MRIs and TVS videoclips and 3D volumes were retrospectively assessed by four experienced radiologists and five experienced sonographers, respectively. Each rater subjectively evaluated the presence or absence of adenomyosis, as well as imaging features suggestive of adenomyosis. Fleiss kappa (κ) was used to reflect inter-rater agreement for categorical data, and the intraclass correlation coefficient (ICC) was used to reflect the reliability of quantitative data. Agreement between raters for diagnosing adenomyosis was higher for TVS than for MRI (κ = 0.42 vs. 0.28). MRI had a higher inter-rater agreement in assessing wall asymmetry, irregular junctional zone (JZ), and the presence of myometrial cysts, while TVU had a better agreement for assessing globular shape. MRI showed a moderate to good reliability for measuring the JZ (ICC = 0.57–0.82). For TVS, the JZ was unmeasurable in >50% of cases, and the remaining cases had low reliability (ICC = −0.31–0.08). We found that inter-rater agreement for diagnosing adenomyosis was higher for TVS than for MRI, despite the fact that MRI showed a higher inter-rater agreement in most specific features. Measurements of JZ in the coronal plane with 3D TVS were unreliable and thus unlikely to be useful for diagnosing adenomyosis.

## 1. Introduction

Adenomyosis is a benign uterine disease characterized by the presence of ectopic endometrial glands and stroma surrounded by hypertrophic myometrium [1]. The symptomatology of adenomyosis includes heavy menstrual bleeding and pelvic pain [2,3,4]. Traditionally, the diagnosis of adenomyosis was only possible through histological examination after hysterectomy. However, advancements in medical imaging techniques, specifically magnetic resonance imaging (MRI) and transvaginal ultrasonography (TVS), have enabled the non-invasive diagnosis of adenomyosis.

MRI has been used as a diagnostic tool for adenomyosis, with the increased thickness of the junctional zone (JZ) with a suggested cut-off of 12 mm [5], and the presence of myometrial cysts is a common criterioin [6]. A principal limitation of MRI is the absence of a definable junctional zone on imaging, which occurs in 20% of premenopausal women [5].

Later studies have suggested additional features, such as an irregular appearance of the JZ, and its relationship with the thickness of the entire myometrium [7,8,9,10]. Another diagnostic feature is the presence of small punctate cystic foci located within the JZ [7,11]. The diagnosis of adenomyosis by MRI has been reported to be highly reproducible [9,12], with high inter-rater agreement for various JZ measurements [9].

TVS, on the other hand, utilizes two-dimensional (2D) and three-dimensional (3D) imaging techniques to assess adenomyosis. The sonographic features used for diagnosing adenomyosis are described well using TVS [13,14,15,16]. A recent consensus paper by the MUSA (Morphological Uterus Sonographic Assessment) consortium distinguishes between direct features of adenomyosis [17]. Direct features include myometrial cysts, hyperechogenic islands, and echogenic subendometrial lines and buds, indicating the presence of ectopic endometrial tissue in the myometrium, and indirect features, which features reflecting changes in the myometrium secondary to the presence of endometrial tissue in the myometrium, i.e., globular uterus, asymmetrical myometrial thickening, fan-shaped shadowing, translesional vascularity, irregular junctional zone, and interrupted junctional zones [17]. The JZ is poorly visualized with 2D TVS. Three-dimensional (3D) TVS offers the possibility of assessing the coronal plane and of using VCI (Volume Contrast Imaging, i.e., thick slice) to improve visualization of the JZ, which facilitates the evaluation of its thickness and irregularity [14,18,19]. Reproducibility studies have shown a high level of agreement when evaluating the presence or absence of adenomyosis using TVS [17,20,21,22] but less agreement when assessing different ultrasound features of the disease [20,21].

The aim of this study was to compare the inter-rater agreement of TVS with MRI regarding the diagnosis of adenomyosis and regarding the assessment of predefined imaging features. By assessing both modalities in the same set of women, we aim to provide insights into their respective diagnostic capabilities and agreement levels.

## 2. Materials and Methods

### 2.1. Study Design and Eligibility Criteria

This prospective study includes consecutive fertile women with heavy regular menstrual bleeding and suspected adenomyosis on clinical examination (including TVS), who were recruited in a private gynecologic clinic in Stockholm. All women were examined and recruited by the same gynecologist (JA) between January 2014 and December 2016 and referred to an MRI and expert TVS as part of the diagnostic workup. Women with adenomyoma, known endometriosis, uterine leiomyomas >4 cm, multiple leiomyomas (>3), or the current use of intrauterine devices or hormonal contraception were not eligible for the study.

In total, 67 women were recruited and referred to MRI and TVS. MRI was missing in 3 women (declined examination, *n* = 2; wrong identification number, *n* = 1) and TVS in 13 women (images were not pseudonymized, *n* = 2; 3D volumes, *n* = 6; or 2D video sequences, *n* = 5, were not recorded or of insufficient quality). Both MRI and TVS images were available in 51 women and included in the study. After the patient recruitment was finalized, the MRIs and TVS videoclips and the 3D volumes were retrospectively assessed by four experienced radiologists and five experienced sonographers, respectively. The assessment of the images was performed between December 2019 and February 2020. The raters were blinded to the clinical history, physical examination, and the evaluation of MRI and TVS images made by other raters.

### 2.2. MRI Examination and Assessment

MRIs of the pelvis were performed in an outpatient facility on a 1.5 T system (Optima MR450w, GE Healthcare, Waukesha, WI, USA, or Siemens Magnetom Symphony Tim, Siemens Healthineers, Erlangen, Germany). The minimum protocol included the following sequences: T2-weigthed Fast Relaxation Fast Spin Echo (FRFSE) or Turbo Spin Echo (TSE) in the axial, sagittal and coronal plane (slice thickness 4–5 mm; gap: 10–20%); T1-weigthed Fast Spin Echo (FSE) or a Gradient Echo (GRE) in the axial and coronal plane (slice thickness 5 mm; gap 10–20%). All examinations were performed with a phased array coil. The women were asked to fast for 4 h before the examination. Antispasmodic drugs were not administered.

**Figure 1:**
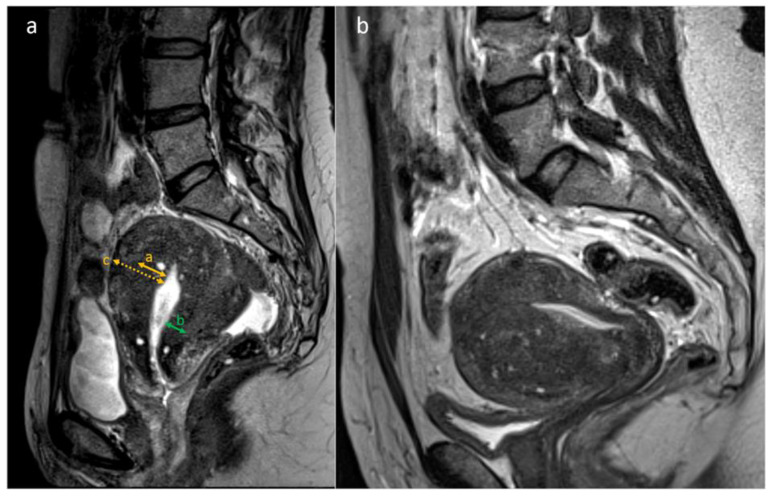
(**a**,**b**). MRI features of adenomyosis: T2-weighted sagittal images from two different subjects with adenomyosis. (**a**). JZmax, the thickest part of the JZ in the midsagittal plane (orange arrow). JZmin, the thinnest part of the JZ in the midsagittal plane (green arrow). Myometrial thickness, measured at the same level as JZmax (orange dotted arrow). (**b**). Example of a uterus with globular shape and asymmetric thickening of JZ (anterior JZ thicker than posterior JZ). The JZ is irregular due to the presence of multiple hyperintense cystic foci within the thickened JZ.

The images were pseudonymized and evaluated on a Picture Archiving and Communication System (PACS; IDS7 version 21.1, Sectra AB, Linköping, Sweden) at the Karolinska University Hospital by four experienced radiologists. Raters could save their assessments and resume them at a later time to reduce the risk of fatigue. With regard to the presence or absence of adenomyosis, each rater based the assessment on their subjective evaluation of the radiological features. There were no standardized criteria given on when to make the diagnosis. The predefined MRI features assessed are listed in Table 1 and shown in Figure 1.

### 2.3. TVS Examination and Assessment

**Figure 2:**
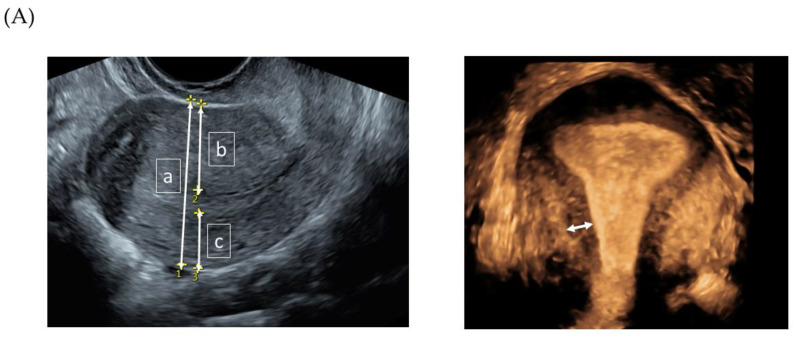

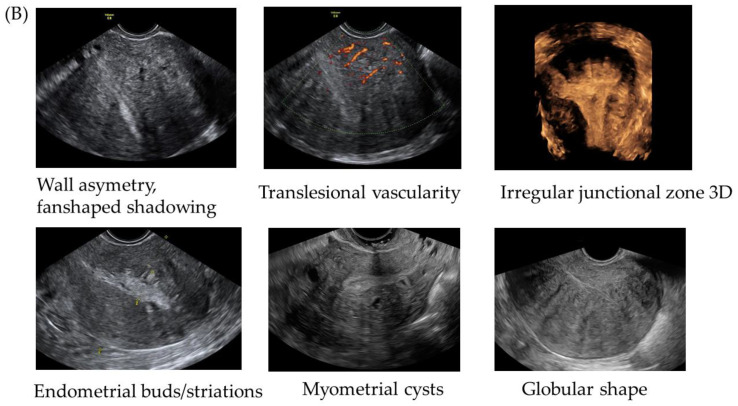
(**A**,**B**). TVS measurements and features in women with adenomyosis. (**A**) Measurement of (a) uterine anterio-posterior diameter, (b) anterior-, and (c) posterior uterine wall thickness in the longitudinal plane. Junctional zone (white arrow), measurement inthe 3D reconstructed coronal plane. (**B**) Examples of sonographic features of adenomyosis.

All women underwent ultrasound examination by a single expert examiner (EE) at the Karolinska University Hospital using a high-end ultrasound system Voluson E10 or E8, GE Healthcare (GE Medical Systems, Zipf, Austria) with a 5–9 MHz 3D transvaginal probe. Two-dimensional grayscale videoclips and 3D-VCI grayscale volumes including the whole uterine body were saved. The GE 4D View software (GE Healthcare, Wood Dale, IL, USA) was used to assess the 3D-VCI volumes. The pseudonymized videoclips and 3D volumes for each case were downloaded to memory sticks and sent to five experienced ultrasonographers. The raters used their own personal computers to assess the volumes. They were encouraged to use high-resolution computer screens and to perform the assessments in a dark room to avoid glare on the screen. The raters could save their assessments and resume later to reduce the risk of fatigue. The volumes were saved in the VCI format with a 2 mm thickness and with a grey mix of 70% X-ray/30% surface smooth. The raters could modify the volume during the analysis to optimize the assessment (remove the VCI function, change slice thickness or grey mix, and rotate the volume in any plane). With regard to the presence or absence of adenomyosis, each rater based the assessment on their subjective evaluation of different ultrasonographic features using pattern recognition [13]. The measurement of the anterior and posterior wall thickness was carried out in a longitudinal plane and the measurement of the junctional zone (JZmax and JZmin) in the reconstructed coronal plane from the 3D volume. The predetermined TVS features assessed are listed in Table 2 and shown in Figure 2A,B.

### 2.4. Statistical Analysis

Each rater entered the assessments into the Research Electronic Data Capture (REDCap^®^, Vanderbilt University) data entry and management program [23,24] hosted at the Karolinska Institutet. REDCap is a secure, web-based software platform designed to support data capture for research studies, providing automated export procedures for data downloads to common statistical packages. Statistical Data Analysis was performed using the Software (SPSS), version 26, IBM Corporation, Armonk, NY, USA.

Fleiss kappa (κ) was used as a measure of inter-rater agreement for categorical data. Kappa values were categorized as “Poor” (κ ≤ 0.20), “Fair” (0.21–0.40), “Moderate” (0.41–0.60), “Good” (0.61–0.80), and “Very good” (0.81–1.00) [25].

The intraclass correlation coefficient (ICC) was used as a measure of reliability for quantitative data, categorized as “Poor” (≤0.5), “Moderate” (0.5–0.75), “Good” (0.75–0.9) and “Excellent” (>0.9), and their 95% confident intervals were calculated based on individual-rating, absolute-agreement, and the 2-way random effects model [26,27].

## 3. Results

The inter-rater agreement results for diagnosing adenomyosis and for the individual imaging features are presented in Table 3 and Table 4.

The five raters of ultrasound images classified adenomyosis as present in 51%, 49%, 39%, 37%, and 76% of the cases, and the four raters of MRI classified the disease as present in 88%, 43%, 49%, and 61%, respectively.

The inter-rater agreement (presented as kappa, i.e., κ = , with [95% Confidence Intervals CI]) for diagnosis of adenomyosis was higher for TVS (κ = 0.42, [0.417–0.422]) than for MRI (κ = 0.28, [0.28–0.287]). For MRI, the inter-rater agreement was moderate for wall asymmetry and irregular JZ and poor for globular uterine shape. For TVS, the inter-rater agreement was moderate for wall asymmetry and globular uterine shape, fair for irregular JZ, fan-shaped shadowing, subendometrial buds and lines, and hyperechogenic islands.

The assessment of cysts in the JZ/myometrium had fair agreement in MRI, and cysts in the myometrium had poor agreement in TVS (κ = 0.327, [0.324–0.331] vs. κ = 0.192, [0.19–0.195]).

The reliability for JZmax was good (ICC = 0.821, [0.709–0.898]) for MRI. For TVS, the JZmax was classified as “not assessable” by one or more rater in 29/51 cases, and ICC was unreliable in the remaining 22 cases (ICC = 0.082, [−0.11–0.252]).

For other continuous data assessed with MRI, ICC showed good reliability for myometrial thickness and JZ differential (JZdiff) and a moderate reliability for JZmin and Ratio JZmax/Myometrium.

The raters of MRIs considered the quality of the images to be medium to high in 47%, 90%, 88%, and 69% of the cases (Table 5), while raters of TVS volumes and video clips considered medium to high quality in 82%, 86%, 67%, 76%, and 88% of the cases (Table 6). In 71%, 94%, 98%, and 80% of the MRI cases (Table 5) and 96%, 94%, 74%, 80%, and 78% of the TVS cases (Table 6), the raters were moderately or highly confident in their evaluation.

## 4. Discussion

We found that the inter-rater agreement for diagnosing adenomyosis was higher for TVS than for MRI, despite the fact that the inter-rater agreement for most individual imaging features was higher for MRI than for TVS. Moreover, MRI showed clearly higher reliability than TVS for continuous variables. Since MRI had a higher agreement for most individual images’ features that were assessed by both TVS and MRI, it is remarkable that the inter-rater agreement for diagnosing adenomyosis was lower for MRI than for TVS. The lack of standardized criteria for diagnosing adenomyosis for MRI is the most likely reason. Since ICC for JZmax was good, the agreement for diagnosing adenomyosis with MRI may have improved if a cut-off for JZ max had been used as the criterion. However, using the JZ as the only criterion for adenomyosis is questioned [9,10]. Also, the absence of cysts may have affected the result. Intramyometrial cysts are pathognomonic for the disease but are found in only one-third to half of affected women [7,8,10]. Some of the participating radiologists may have excluded the diagnosis when no cysts were found, even when altered JZ features were present. The limited agreement for cysts may have negatively affected the agreement for diagnosis. A recent meta-analysis and review on the performance of various objective criteria diagnosing adenomyosis, using MRI, concluded that most parameters have a relatively low sensitivity and a relatively high specificity [28]. JZ characteristics remain the most widely used and investigated with acceptable diagnostic accuracy. Specific research is needed into how these objective measures of adenomyosis can be correlated to clinical outcomes.

For TVS, the inter-rater agreement was fair or moderate for most of the individual ultrasound features and moderate for diagnosing adenomyosis. This is in line with the results of other studies [18,20] showing a good reproducibility in the diagnosis of adenomyosis using 2D TVS pattern recognition. When an ultrasound diagnosis of adenomyosis is made using pattern recognition, all the different ultrasound features are taken into account. This may explain why agreement regarding diagnosis was higher than agreement for most individual features. When combined, the subjective overall agreement for adenomyosis is present or not becomes higher than the agreement for various variables. The inter-rater agreement for cysts in the myometrium was poor. Small cysts in the myometrium may be difficult to detect with TVS, especially when other features, such as shadowing, are present. In this study, the globular uterus shape and wall asymmetry showed the highest agreement between raters, whereas other studies have reported the highest agreement for irregular JZ [20,21].

With 3D TVS, it is possible to visualize the JZ. However, JZ measurements with 3D TVS have been shown to have limited reproducibility [20,21,22]. In our study, there were missing data from >50% of the TVS cases since cases were excluded from the ICC analysis if one rater classified the JZ as “not assessable”, even though the other raters measured the JZ. Moreover, results were unreliable in the cases where all the TVS raters measured the JZ, indicating low diagnostic value in clinical practice. One reason may be the low quality of 3D volumes in women with adenomyosis, as the abundant scattering of the ultrasound beam results in poor image quality in the reconstructed coronal plane, making assessment difficult. Moreover, the coronal plane may not be the correct plane to measure JZ. A recent update to the MUSA consensus paper suggests assessing the regularity of the junctional zone in multiple planes (transversal, longitudinal, coronal) using 3D ultrasound, since a regular JZ can rule out adenomyosis, while measurements of the JZ was dismissed because of a lack of evidence of the clinical relevance of this measurement [29].

A recent meta-analysis showed that MRI and TVS had an adequate performance with regard to diagnosing adenomyosis, with a pooled sensitivity and specificity of 75% and 81% for TVS and 69% and 80% for MRI, *p* = 0.75, when hysterectomy was used as a gold standard [30]. Still, both modalities have shown unsatisfactory inter-rater agreement for the diagnosis, indicating that adenomyosis is challenging even for experts. Clear definitions and criteria, along with more standardized interpretation models for imaging are needed for both MRI and TVS to make the diagnosis more reliable.

A strength of the study is the large number of cases examined with both imaging modalities and the enrollment of raters from different centers. For TVS, expert raters from different centers in Europe were included, which increases the generalizability of the study. Unfortunately, this was not possible for MRI.

Although the TVS images were assessed offline and a dedicated “state-of-the-art” MRI was not applied, the majority of raters considered the quality of the images to be medium or good and were confident in their evaluation. Although the image quality was suboptimal, it still appears acceptable for assessing the required parameters and reflects the reality in a clinical set-up.

The use of the RedCap database reduced human error in the handling of data to a minimum and ensured complete datasets. All questions were mandatory and thus had to be answered before the raters moved on to the next case. Missing data were only present for JZ measurements, in cases where it was not possible to identify the JZ, and thus it was classified as “not assessable” and consequently not measurable.

A limitation of the study is the use of stored offline TVS videos and volumes instead of real-time examinations. It is well known that 2D TVS image quality is often reduced by the presence of adenomyosis, as it gives rise to shadowing and distorts the normal endometrial–myometrial border. The effect of poor image quality in the 3D rendering plane further reduces the quality in the reconstructed coronal plane, hampering assessment in low-quality 3D volumes. However, it would be impossible to carry out reproducibility studies with multiple raters in TVS without using recorded material. Even for MRI, the quality of the images was not optimal. The examinations were performed in a private radiological outpatient center where neither an abdominal belt nor an antispasmodic agent was used to reduce motion artefacts caused by small-bowel peristalsis. Several MRIs were affected by artefacts, thus hampering the quality of the images. However, consensus guidelines suggesting technical protocols for MR imaging of endometriosis were published after the enrollment of the study subjects [31,32]. Furthermore, no oblique axial T2-weighted sequence perpendicular to the long uterine axis was included in the MRI protocol, which is useful for the assessment of adenomyosis [8,10]. Finally, it is important to point out that we included consecutive women with a clinical suspicion of adenomyosis, representing a real-life setting where the histological outcome was not available in the majority of women, as they underwent medical treatment. We find it acceptable not to have a gold standard for comparison, as our aim was to assess inter-rater agreement and not to correlate TVS and MRI to histology.

## 5. Conclusions

The inter-rater agreement for diagnosing adenomyosis was higher for TVS than for MRI despite MRI manifesting higher inter-rater agreement in most variables, in particular variables related to measuring the junctional zone. The measurement of JZ thickness in the coronal plane with 3D TVS could only be performed in fewer than half of the women and was found to be unreliable in the rest, and it is therefore unlikely to be useful for diagnosing adenomyosis.

## Figures and Tables

**Table 1:** MRI features included in the assessment.

JZ ^1^ max	Maximal JZ thickness (mm). Thickest part of the JZ in the midsagittal plane.
JZmin	Minimal JZ thickness (mm). Thinnest part of the JZ in the midsagittal plane.
Myometrial thickness	The thickness (mm) of the uterine wall at the same level as JZmax.
JZdiff	JZ differential (mm). Difference between the JZmax and JZmin.
JZmax/Myometrial thickness	The extent of the uterine wall that is affected by adenomyosis (%), JZmax divided by myometrial thickness measured at the same level
Appearance of JZ	(a) Not visible or not assessable. (b) Regular JZ: the inner and outer borders of the JZ are well defined. (c) Irregular JZ; the presence of focal thickening of the JZ and/or presence of cystic foci of high signal intensity.
Cysts	Cystic changes in the JZ (defined as foci of high signal intensity on T2-weighted images). Absent/Present.
Globular uterine shape	Subjective evaluation of the uterine corpus being globular shaped and not due to fibroids. Globular yes/no.
Wall asymmetry	Subjective evaluation of the anterior and posterior wall thickness. Yes/no.
Diagnosis	Assessment of the images. Does the subject have adenomyosis? Yes/No
Image Quality	Evaluation of the quality of the images. Low/Moderate/High
Diagnostic confidence	Examiner confidence in their assessment. Low/Medium/High

^1^ Junctional zone (JZ).

**Table 2:** TVS features included in the assessment.

JZ ^1^ max	Maximal JZ thickness (mm), measured in the reconstructed coronal plan from the 3D ^2^ volume.
JZmin	Minimal JZ thickness (mm), measured in the reconstructed coronal plane from 3D ^2^ volume.
Globular uterine shape	Subjective evaluation of the uterine corpus being globular-shaped and not due to fibroids. Yes/No.
Wall asymmetry	Subjective evaluation of the anterior and posterior wall thickness. Are the walls asymmetric? Yes/No
Appearance of JZ	Assessment of the JZ irregularity. Regular/Irregular
Fan-shaped shadows	Is fan-shaped shadowing present in the myometrium? Yes/No.
Buds/Striations	Are endometrial buds/striations present in the myometrium? Yes/No
Hyperechogenic Islands	Are hyperechogenic islands present in the myometrium? Yes/No
Cysts	Presence of myometrial cysts? Yes/No
Diagnosis	Assessment of the images. Does the subject have adenomyosis? Yes/No.
Quality	Evaluation of the quality of the images. Low/Moderate /High
Confidence	Examiner confidence in the assessment. Low/Medium/High

^1^ Junctional zone (JZ), ^2^ Three-dimensional (3D).

**Table 3:** Interrater agreement for categorical data presented with Fleiss kappa.

Variable	MRI		TVS	
	Kappa	95% CI	Kappa	95% CI
Globular uterus shape	0.199	0.195–0.202	0.482	0.48–0.485
Wall asymmetry	0.552	0.548–0.556	0.414	0.411–0.417
Irregular JZ ^1^	0.527	0.522–0.531	0.309	0.307–0.312
Cysts in the JZ/Myometrium	0.327	0.324–0.331	0.192	0.190–0.195
Fan-shaped shadowing	-	-	0.357	0.354–0.359
Buds or Striations	-	-	0.209	0.206–0.211
Hyperechogenic Islands	-	-	0.289	0.286–0.292
Diagnosis	0.283	0.280–0.287	0.420	0.417–0.422

^1^ Junctional zone (JZ).

**Table 4:** Interrater reliability for continuous data presented with intraclass correlation, ICC.

Variable	MRI		TVS	
	ICC	95% CI	ICC	95% CI
JZ ^1^ max	0.821 ^2^	0.709–0.898	0.082 ^3^	−0.11–0.252
JZmin	0.572 ^2^	0.408–0.724	−0.31 ^4^	−0.67–−0.104
JZmax/Myometrial thickness	0.830 ^2^	0.739–0.900	-	-
RatioJZmax/Myometrium	0.570 ^2^	0.382–0.731	-	-
JZmax-JZmin (JZdiff)	0.713 ^2^	0.583–0.823	-	-

^1^ Junctional zone (JZ); ^2^ Missing value: 15 cases are excluded since at least one rater evaluated JZ as “not assessable”; ^3^ Missing value: 29 cases are excluded since at least one rater evaluated JZ as “not assessable”; ^4^ Missing value: 42 cases are excluded since at least one rater evaluated JZ as “not assessable”.

**Table 5:** MRI. Assessment of image quality and confidence in evaluation.

	Rater 1				Rater 2				Rater 3				Rater 4			
	Quality		Confidence		Quality		Confidence		Quality		Confidence		Quality		Confidence	
	*N*	%	*N*	%	*N*	%	*N*	%	*N*	%	*N*	%	*N*	%	*N*	%
Low	27	52.9	15	29.4	5	9.8	3	5.9	6	11.8	1	2.0	16	31.4	10	19.6
Medium	18	35.3	19	37.3	18	35.3	17	33.3	21	41.2	10	19.6	30	58.8	27	52.9
High	6	11.8	17	33.3	28	54.9	31	60.8	24	47.1	40	78.4	5	9.8	14	27.5

**Table 6:** TVS. Assessment of image quality and confidence in evaluation.

	Rater 1				Rater 2				Rater 3				Rater 4				Rater 5			
	Quality		Confidence		Quality		Confidence		Quality		Confidence		Quality		Confidence		Quality		Confidence	
	*N*	%	*N*	%	*N*	%	*N*	%	*N*	%	*N*	%	*N*	%	*N*	%	*N*	%	*N*	%
Low	9	17.6	2	3.9	7	13.7	3	5.9	17	33.3	13	25.5	12	23.5	9	17.6	6	11.8	11	21.6
Medium	35	68.6	7	13.7	35	68.6	33	64.7	21	41.2	12	23.5	17	33.3	9	17.6	37	72.5	24	47.1
High	7	13.7	42	82.4	9	17.6	15	29.4	13	25.5	26	51.0	22	43.1	32	62.7	8	15.7	16	31.4

## Data Availability

Database available on request; images not available.
